# Scalable Fabrication of High-Performance Transparent Conductors Using Graphene Oxide-Stabilized Single-Walled Carbon Nanotube Inks

**DOI:** 10.3390/nano8040224

**Published:** 2018-04-07

**Authors:** Linxiang He, Chengzhu Liao, Sie Chin Tjong

**Affiliations:** 1 Department of Physics, City University of Hong Kong, Tat Chee Avenue, Kowloon, Hong Kong, China; linxiang_he@hotmail.com; 2 Department of Materials Science and Engineering, Southern University of Science and Technology, Shenzhen 518055, China

**Keywords:** aqueous dispersion, carbon nanotube, graphene oxide, ink, rod coating, electrical conductivity, optical transmittance, mechanical flexibility

## Abstract

Recent development in liquid-phase processing of single-walled carbon nanotubes (SWNTs) has revealed rod-coating as a promising approach for large-scale production of SWNT-based transparent conductors. Of great importance in the ink formulation is the stabilizer having excellent dispersion stability, environmental friendly and tunable rheology in the liquid state, and also can be readily removed to enhance electrical conductivity and mechanical stability. Herein we demonstrate the promise of graphene oxide (GO) as a synergistic stabilizer for SWNTs in water. SWNTs dispersed in GO is formulated into inks with homogeneous nanotube distribution, good wetting and rheological properties, and compatible with industrial rod coating practice. Microwave treatment of rod-coated films can reduce GOs and enhance electro-optical performance. The resultant films offer a sheet resistance of ~80 Ω/sq at 86% transparency, along with good mechanical flexibility. Doping the films with nitric acid can further decrease the sheet resistance to ~25 Ω/sq. Comparing with the films fabricated from typical surfactant-based SWNT inks, our films offer superior adhesion as assessed by the Scotch tape test. This study provides new insight into the selection of suitable stabilizers for functional SWNT inks with strong potential for printed electronics.

## 1. Introduction

Recently, there is a strong demand for transparent conductors (TCs) for various optoelectronic devices such as touch screens, solar cells, organic light-emitting diodes (OLEDs), etc. [1,2,3,4]. The main TCs used currently are indium-doped tin oxide (ITO). However, indium is very expensive [5], and almost all ITO films are deposited onto glass substrates through a rather slow sputtering technique, resulting in further rises in production costs. The successful development of single-walled carbon nanotubes (SWNTs) in the 1990s has attracted tremendous scientific attention due to their excellent mechanical, electrical and thermal properties [6]. SWNTs can be fabricated into thin films having high optical transparency and electrical conductivity [7], rendering those ideal alternatives for replacing ITO films. Solution-based approaches such as spray coating [8], spin coating [9], Langmuir-Blodgett technique [10], vacuum filtration [11], dip coating and rod coating [12,13,14], are usually employed to prepare SWNT films. However, as-produced SWNTs generally form agglomerates and are difficult to disperse in water or organic solvents at appropriate concentrations. To effectively disperse them, several strategies, such as chemical modification, surfactants or polymeric dispersant additives, are usually employed [12,13,15,16,17]. As recognized, the dispersants typically used are electrical insulators and therefore must be removed from the resultant films. This is a difficult or tedious task because dispersants used to debundle SWNTs interact strongly with the nanotubes and are hard to remove from them. Furthermore, the additions and subsequent removal of dispersants increase production costs and degrade the mechanical performance of the films greatly [18].

Graphene oxide (GO), an oxygenated graphene sheet, is a low-cost chemically derived graphene prepared by chemical oxidation of graphite flakes in strong oxidizing solutions [19]. GO consists of hydrophobic domains with a benzene ring structure and hydrophilic sites with hydroxyl, carboxyl and carbonyl groups [20]. This unique feature renders GO acting as an amphiphilic molecule and enables it to form Langmuir-Blodgett films on a water surface [21]. The oxygenated moieties make GO sheets hydrophilic and highly dispersible in water, whereas aromatic domains interact with other aromatic molecules through supramolecular π-π interactions [22]. Using GO to disperse SWNTs has a major benefit: GO can be thermally/chemically reduced to form reduced graphene oxide (rGO) [23], which is electrically conductive, thus enhancing the conductivity of resultant films.

Fabrication of GO-SWNT films have been previously reported by several researchers. For example, Tian et al. carried out a preliminary study on the preparation of transparent GO-SWNT films in which aqueous GO-dispersed SWNT solutions were air-sprayed onto glass substrates [24]. Their GOs were not chemically reduced to rGOs, so the GO-SWNT films exhibited a large sheet resistance of ~4 kΩ/sq at 85% transmittance. Zheng et al. prepared GO-SWNT films by means of a layer-by-layer Langmuir-Blodgett assemble process [25]. The resultant films showed a sheet resistance ranging from 180 Ω/sq to 560 Ω/sq at ~77–86% transparency depending on the number of layers. Very recently, Gorkina et al. fabricated GO-SWNT films using several steps. In the process, SWNT film was first deposited onto a substrate via a dry transfer process, then GO was sprayed on top of SWNT film. GO was thermally reduced at ambient or hydrogen atmosphere and finally reacted with AuCl_3_ [26]. They reported that the mean sheet resistance of hybrid SWNT/rGO films treated with air and hydrogen was 485 Ω/sq and 264 Ω/sq, respectively, at about 85% transmittance. All these processes are unsuitable for industrial scale-up production. By contrast, coating SWNT inks to plastic substrates with a Mayer rod to form thin films is considered of technological importance. Fluids that can be coated effectively by a Mayer rod is readily adapted to industrial process, allowing rapid roll-to-roll printing at speeds up to 100 m/min on plastic substrates of several meters wide [27]. Moreover, transparent film fabrication by rod coating of SWNT inks is less wasteful, so nearly all the SWNTs are well deposited on the substrate. However, simple SWNT dispersions cannot be used as inks to fabricate thin films, since their rheological properties and wetting behavior are inappropriate for rod coating practice. Therefore, additives are usually incorporated to improve their viscosity, wetting, levelling, etc. However, typical or commercial additives used for modifying rheological properties and/or wetting behavior can induce instability of the dispersions or adversely affect optoelectronic properties of the resulting films. For example, Dan et al. reported that a dual surfactant system, sodium dodecylbenzenesulfonate (SDBS) and Triton X-100 (TX100), can be used to prepare SWNT inks [13]. Li et al. indicated that this mixed surfactants-SWNT system is stable for only a few minutes, making it unsuitable for industrial applications [28]. As such, more efficient strategies are desired to develop large-scale industrial production of these carbon nanostructures.

In a previous study, we reported the use of GO as a dispersant to prepare multi-walled carbon nanotube (MWNT) inks to generate transparent conductive MWNT films [14]. MWNT-based films, however, generally exhibit poor electro-optical performance than the films prepared from SWNTs [29]. This is because every graphene layer of MWNTs contributes to the light absorption, causing the films with strong light absorption [30]. For this reason, the development of carbon nanotube-based TCs has been primarily carried out with SWNTs. Herein, we report and demonstrate large-scale preparation of SWNT films through a Mayer rod coating of aqueous GO-SWNT inks. By converting GO to rGO through microwave irradiation, these films exhibit much improved electro-optical properties, making them very attractive materials for making various optoelectronic devices.

## 2. Materials and Methods

### 2.1. Materials and Synthesis

Chemical vapor deposited SWNTs (>95 wt % purity; diameter of 0.8~1.6 nm and length of 5~30 μm) (Figure S1) were supplied by Chengdu Organic Chemicals Co. Ltd., Chengdu, China. Graphite flakes were bought from Sigma-Aldrich, Inc., St. Louis, MO, USA. Graphite oxide was prepared from graphite flakes by means of Hummers process [19]. It was readily exfoliated into monolayer GO sheets in water (Figure S2). All chemical reagents were used as received without further purification.

### 2.2. rGO-SWNT Films

Aqueous GO dispersions of different concentrations were obtained by stirring graphite oxide solids in water for 4 h, and the mixtures were then sonicated (Tru-Sweep 575 HTAG, Crest Ultrasonics Corporation, Ewing Township, NJ, USA) for 30 min. To such dispersions SWNTs were added, obtaining a concentration of 0.2 mg/mL SWNTs. This concentration was relatively lower than in previous works to ensure full exfoliation of nanotube bundles, since long nanotubes generally caused higher viscosity and decreased the dispersion efficiency. These GO-SWNT dispersions were then tip sonicated (SCIENTZ-II D Ultrasonic Homogenizer) in an ice-water bath for 2 h. This sonication process reduced the size of GO sheets and the diameter of nanotube bundles, resulting in more homogeneous GO-SWNT dispersions. To prepare films, formulated GO-SWNT dispersions were coated onto polyethylene terephthalate (PET) sheets by a Mayer rod. The thickness of the films was controlled by the diameter of a wire coiled on the rod. To convert GO to rGO, the films were firstly exposed to a hydrazine atmosphere for 1 h to partly recover its conductivity, then treated in a microwave oven (Panasonic NN-ST253W, 800 W, Panasonic Corporation, Osaka, Japan) in an argon atmosphere for 2~3 s. This microwave treatment rapidly heated the films and thermally reduced GO to rGO [31,32].

### 2.3. Material Characterization

The quality of GO-SWNT inks was examined with UV-vis spectroscopy (Agilent Cary 5000 Spectrophotometer, Agilent Technologies Inc., Santa Clara, CA, USA), Raman spectroscopy (Horiba Jobin Yvon LabRAM HR Evolution Micro-Raman spectrometer; excitation wavelength of 532 nm, Horiba Ltd., Kyoto, Japan), transmission electron microscopy (TEM; Philips FEG CM 20, Philips Company, Amsterdam, Netherlands) and zeta potential measurements (Zetaplus, Brookheaven, NY, USA). The contact angle of those inks on PET was obtained with an advanced goniometer (Ramé-Hart Instrument Model 200). The viscosity of the inks was measured using a rheometer (TA Instruments, HR-2 Discovery Hybrid Rheometer, TA Instruments, New Castle, DE, USA). The surface morphology of rGO-SWNT films was observed in both field-emission scanning electron microscope (SEM; Jeol FEG JSM 6335, JEOL Company, Tokyo, Japan) and atomic force microscope (AFM; Veeco Nanoscope V, Veeco Inc., Plainview, NY, USA). We employed X-ray photoelectron spectroscopy (XPS; PHI-5802; Physical Electronics, Chanhassen, MN, USA) with Al K_α_ source radiation to record chemical bonding states of constituent carbon in the films. The electrical sheet resistance of thin films was determined with a Van der Pauw setup using four electrodes aligned along the circumference of thin films. The light transmittance tests at ambient were performed with a UV-vis spectrophotometer (PerkinElmer Lambda 2S, Perkin Elmer Incorporation, Waltham, MA, USA). For bending cycling, samples were bent to a radius of curvature of 2 mm by sliding around a metal rod. In the meanwhile, electrical resistance was measured up to 2000 cycles. For the film adhesion testing, a strip of tape was applied to the sample and pressed down to uniformly contact the film surface, and then peeled off at ~10 mm/s at a ~45° angle. The sheet resistance and film transmittance before and after the tests were measured to determine the film adhesion properties.

## 3. Results

### 3.1. Dispersing SWNTs in Water Using GO

**Figure 1:**
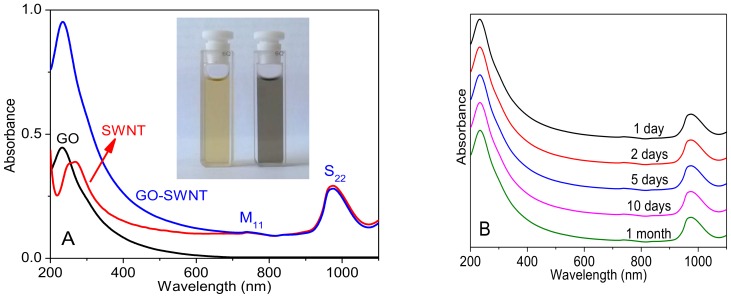

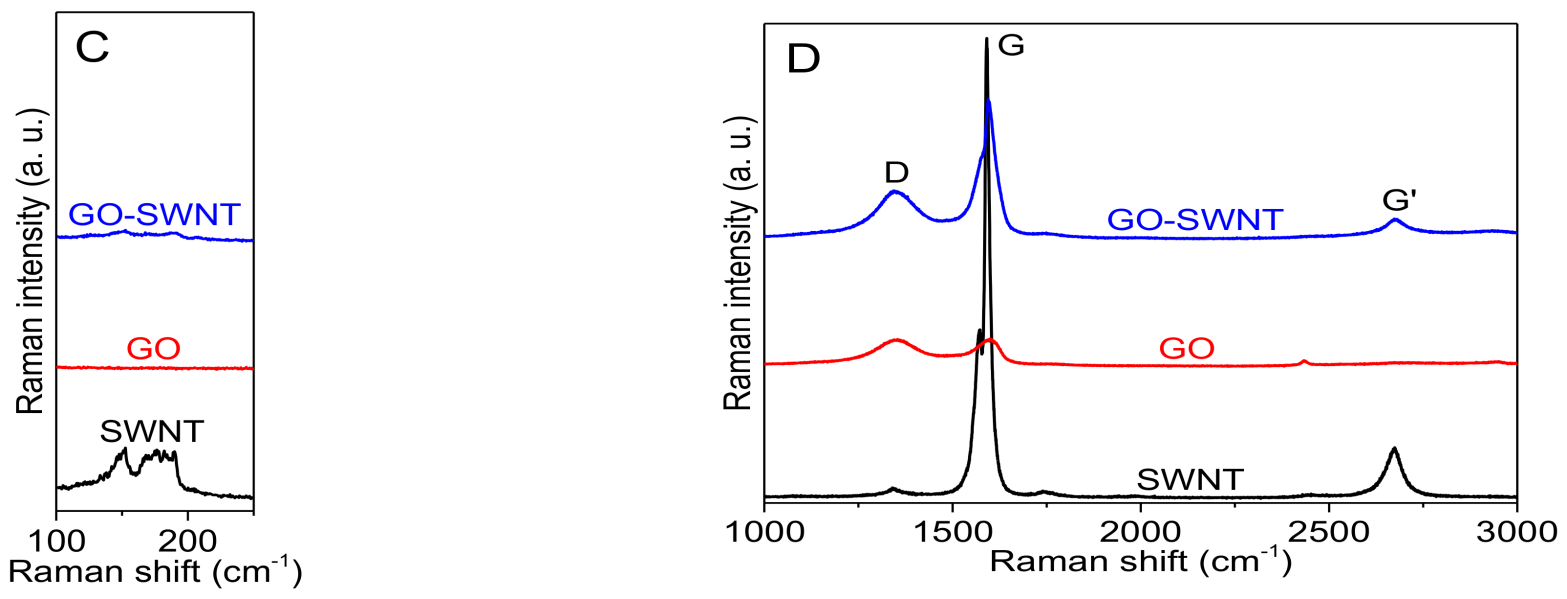
(**A**) UV-vis absorbance of diluted GO and GO-SWNT (weight ratio of 10:1) dispersions. Pure SWNT solution (weakly oxidized by nitric acid) is shown for comparison. The inset photo displays GO-SWNT (left) and GO (right) dispersions. (**B**) Absorbance of GO-SWNT dispersion for different time periods. (**C**,**D**) Raman profiles of GO-SWNT dispersion (weight ratio of 10:1), GO and SWNT.

Attaining well-dispersed SWNTs without aggregates is key to ensure successful fabrication of homogeneous films. Due to strong van der Waals interactions amongst SWNTs, they tend to aggregate to form precipitates in water. At sufficient GO, however, SWNTs can be effectively dispersed. We prepared a series of GO-SWNT dispersions with different GO to SWNT mass ratios, and found that homogeneous dispersions can be obtained only when the mass ratio reaches at least 5, at a 0.2 mg/mL SWNT concentration. By diluting with a factor of ~20, these dispersions look like “true solutions” (Figure 1A). UV-vis spectroscopy was then performed to characterize their absorption characteristics (Figure 1B). The band located at ~1000 nm is the S22 interband transition in semiconducting SWNTs, and the M11 peak at ~750 nm relates interband transition in metallic SWNTs. These dispersions are very stable and no visible sediment is found for several months. Zeta potential measurements on these GO-SWNT dispersions agree reasonably with the absorption spectra findings. Pure GO dispersion at 0.1 mg/mL and neutral pH exhibits a zeta potential of 55 ± 5 mV. For the GO-SWNT dispersion with a high SWNTcontent (0.02 mg/mL), zeta potential remains almost unchanged. The results demonstrate that the GO-SWNT dispersion is very stable, and SWNTs are effectively dispersed without changing original electrostatic repulsions among the GO sheets.

Figure 1C,D display Raman spectra of SWNT, GO and the GO-SWNT dispersion. The radial breathing mode (RBM) is a characteristic property of SWNT, i.e., diameter of SWNT is inversely proportional to its RBM peak frequency. Figure 1C reveals the presence of SWNT multi-peaks, so there exists a fairly wide distribution of SWNT diameters. These peaks are significantly suppressed in hybrid GO-SWNT spectrum, demonstrating that GO sheets interact with the nanotubes and severely limit their RBM vibration [33]. From Figure 1D, the G-band at ~1590 cm^−^^1^ is due to the vibrational modes of sp^2^ carbon. The D-band at ~1350 cm^−^^1^ is Raman active with its value proportional to the amount of amorphous carbon. The G-band of GO is significantly weaker than SWNT, and its D-band is reasonably more intense. The G-band of the GO-SWNT hybrid is dominated by SWNT because no absorption occurs for GO sheets at the excitation wavelength and thus no resonance enhancement [25]. On the other hand, the D-band in the hybrid is disproportionally higher, probably due to the contribution of GO sheets in combination with the effects similar to those displayed by the functionalized nanotubes [24].

**Figure 2:**
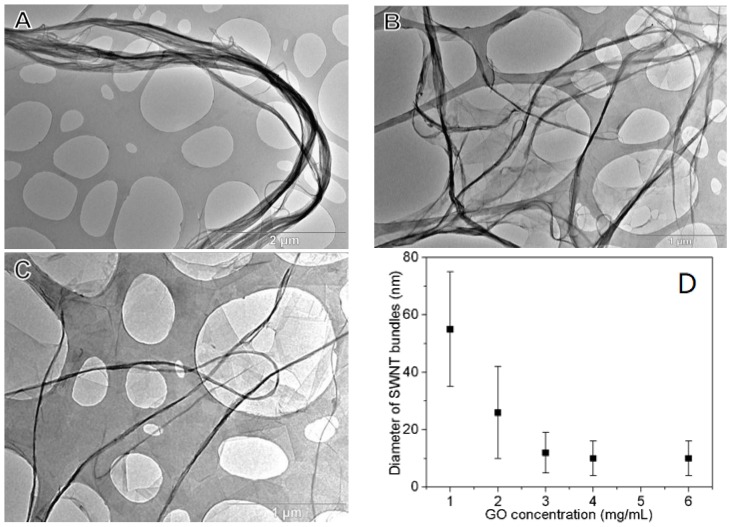
TEM micrographs of (**A**) SWNT bundles in water; (**B**) GO-SWNT dispersion with a GO:SWNT mass ratio of 10:1; (**C**) GO-SWNT dispersion with a GO:SWNT mass ratio of 20:1. The concentration of SWNT in each case is 0.2 mg/mL; (**D**) Effect of GO concentration on the diameter of SWNT bundles in the dispersion. The SWNT content in the ink is 0.2 mg/mL.

The dispersion state of SWNTs was further imaged by TEM as shown in Figure 2. As recognized, pristine SWNTs tended to agglomerate into thick ropes (Figure 2A). In the presence of GO, however, these ropes were exfoliated into thinner ones (~40 nm), and separated from one another by adhering to GO (Figure 2B), showing a strong interaction between GO and SWNT. By increasing the amount of GO, the diameter of the SWNT ropes become thinner. At a mass ratio of 20, these ropes decreased to ~15 nm in diameter (Figure 2C). Due to the huge aspect ratio of SWNTs (length of 5~30 μm and diameter of 0.8~1.6 nm), very strong interactions among them is expected. Therefore, further exfoliation of the SWNT ropes is not possible even in the presence of excessive GO. Figure 2D summarizes the effect of GO concentration on the average diameter of SWNT bundles in the dispersion. These results reveal the successful dispersion of SWNTs with GO. Furthermore, the dispersion is stable during drying and this is crucial for the film fabrication.

Together, these results indicate that GO can effectively debundle SWNTs into individual or thin nanotubes. The energy required for dispersing SWNTs in water is large, because of its huge specific surface area and nonpolar surface behavior, thereby inducing aggregation of SWNTs in water (Figure 2A,B). Two mechanisms can be proposed for good nanotube dispersion with GO. The first one relates to the π-π interaction between GO and SWNT. It is known that GO has lots of π-conjugated aromatic domains within its basal plane, therefore it can interact strongly with SWNTs via π-π attractions [34]. From the literature, drug and dye molecules can adsorb onto the surface of GO through π-π interactions [34]. Besides, it is widely recognized that GOs are strongly hydrated by dispersing in water. When the nanotubes adsorb on GOs, all water molecules attached to the GOs are released, leading to a significant gain in entropy [35]. Thus the crucial factor for the effective dispersion of SWNTs by GOs arises from π-π interactions between the nanotubes and GOs. From the TEM images it is clear that GOs interact with SWNTs by anchoring SWNT bundles. At low GO content (GO:SWNT mass ratio of 10:1), the number of aromatic sites available for anchoring SWNTs is rather low, so thick nanotube bundles tend to form. In the presence of sufficient GOs (GO:SWNT mass ratio of 20:1), these bundles can be effectively exfoliated, resulting in thinner nanotubes. The demonstrated procedure of using GOs to disperse SWNTs in water offers the possibility of low cost and large-scale preparation of excellent SWNT dispersions.

### 3.2. Wetting Behavior and Rheological Properties of GO-SWNT Dispersions

**Figure 3:**
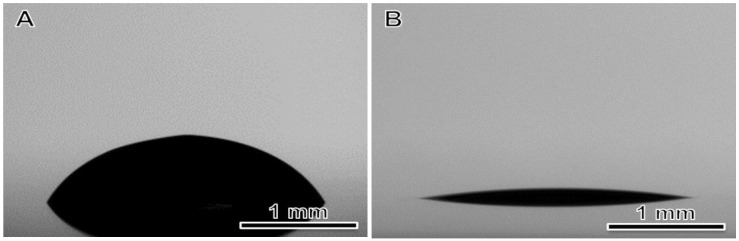
Contact angles for GO-SWNT ink on PET substrate: (**A**) before and (**B**) after plasma treatment. The contact angle is 59.7° and 10.2°, respectively.

Rod coating involves the spreading of liquid drops to form a thin liquid layer on the substrate. Contact angle is usually used to evaluate the wettability of a solid surface by a liquid. Large contact angle (larger than 90°) reflects poor wettability. Moreover, the spreading of the liquid causes a significant increase in its surface area, resulting in a steep rise of its surface energy. To minimize the surface energy the wet film would contract its surface accordingly. This often causes film nonuniformities such as contact line recession, dewetting or film rupture. To avoid these nonuniformities (and therefore minimize film contraction), a small surface tension is preferred. The surface tension of GO-SWNT inks and their contact angle with the PET substrate were determined with a goniometer (Figure S3), and were observed to be nearly independent of ink compositions, with σ ~ 70 mN/m and θ ~ 60°, respectively. The relatively larger contact angle indicated a relatively poor wetting of the GO-SWNT ink on PET. To improve the wetting behavior, we treated the PET substrate with oxygen plasma for ~10 min. This decreased the contact angle to ~10° (Figure S4 and Figure 3B).

Immediately after rod coating, the wet film exhibits a wavy surface in the form of stripes with their interspace distances governed by the diameter of coiled wire. To obtain a flat and smooth surface these stripes must be levelled off. For thin films this leveling process is motivated by the difference in surface tension between the convex and concave regions of wavy liquid. However, the viscous forces play a part in retarding this process. The leveling time is defined as *t*_level_ = 3 µr^4^/σh^3^ [36], where µ is the viscosity of the ink, 2πr is the diameter of coiled wire, and h is the average wet thickness. For liquids like GO-SWNT inks, leveling can therefore be accomplished by reducing the viscosity. During the drying process, dewetting of the wet film may occur, resulting in film nonuniformities as mentioned before. The dewetting time is *t*_dewet_ = µL/kσθ^3^ [37], where *l* is the characteristic length scale of the film and k relates to a fluid property, being about 10^−3^ for primarily water-based system. Therefore, dewetting can be suppressed by reducing the contact angle θ, and/or by increasing the viscosity µ. Since the preparation of a uniform film requires a fast leveling process (small *t*_level_) and a slow dewetting process (large *t*_dewet_), optimization of the ink viscosity is considered of primary importance.

**Figure 4:**
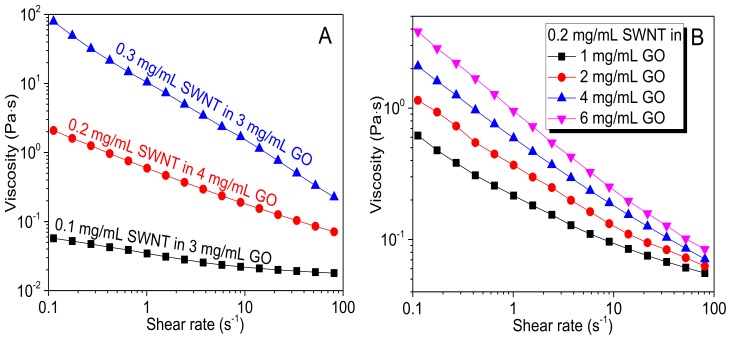
Effects of the concentration of (**A**) SWNT and (**B**) GO on the viscosity of GO-SWNT ink.

Inks generally exhibit the non-Newtonian shear-thinning property. At the film formation stage, inks are seriously sheared through the grooves of the rod, thereby flowing at a high-shear rate. During the drying process, they are nearly stationary and flow at a low-shear rate. For a typical rod coating setup, the optimal viscosity value falls in 0.01–1 Pa s range [38]. The characteristic shear rate for the film formation region is ~50 s^−1^ in the present study. Hence, the optimal viscosity value of the GO-SWNT ink should fall in 0.01–1 Pa s range and near 50 s^−1^. Figure 4 shows that all GO-SWNT inks exhibit a drop in viscosity under shear. This is a thixotropic behavior since the viscosity decreases with shear rate. Such behavior is also displayed by other non-Newtonian fluids such as polymer solutions and biological fluids [39,40,41,42,43,44]. For GO-SWNT dispersions, this behavior derives from debundle of nanotubes and/or by the increased orientation of nanotubes in the flow direction. Both the SWNT and GO concentration are found to influence the viscosity. Since the nanotubes used in this study have very large aspect ratios, the nanotube concentration would play a dominant role in determining the viscosity [45]. Doubling its concentration from 0.1 mg/mL to 0.2 mg/mL can increase both low-shear viscosity by about one order of magnitude (~2 Pa s at ~0.1 s^−1^), and high-shear viscosity by about 5-fold (~85 Pa s at ~50 s^−1^). Increasing the concentration to 0.3 mg/mL would lead to a dramatic increase in low-shear viscosity by about two orders of magnitude (~100 Pa s at ~0.1 s^−1^), which is high enough to enable the inks to be used for other coating methods like blade coating or screen coating. In contrast, increase the GO concentration from 1 mg/mL to 6 mg/mL (at 0.2 mg/mL SWNT) only slightly increases the viscosity, as shown in Figure 4B. Inks with 0.2 mg/mL SWNTs are found to fall into the optimal viscosity range (0.01–1 Pa s) at a shear rate between 0.1 s^−1^ and 50 s^−1^, therefore are most suitable for rod coating.

Mayer rod coating of optimized GO-SWNT dispersions generally produces homogeneous defect-free films. The thickness of these films can be controlled by simply choosing coiled rods of different sizes. In this study, the diameter of wire coiled on the rod ranges from 0.2 mm to 2 mm, resulting in wet thicknesses of 20–200 µm and final dry thicknesses of ~10–100 nm.

Table 1 summarizes different time parameters involved in the fabrication of TCs using GO-SWNT inks with different compositions. *t*_level_ is determined based on the high-shear viscosity, as the fluid close to the moving rod is highly sheared while being coated. It lies in the order of 10^−3^ s, indicating that levelling occurs immediately after coating to form a flat wet film; *t*_dewet_ is calculated using the low-shear viscosity, since the wet film is almost stationary during the drying process. It lies in the order of 10^3^ s, which is much larger than *t*_dry_, implying that these films can dry before secondary flow occurs. The relationship *t*_level_ < *t*_dry_ < *t*_dewet_ maintains for all these GO-SWNT dispersions, indicating that they can be employed as inks to prepare films with uniform thickness and limited dewetting.

### 3.3. Fabrication & Characterization of rGO-SWNT Films

**Figure 5:**
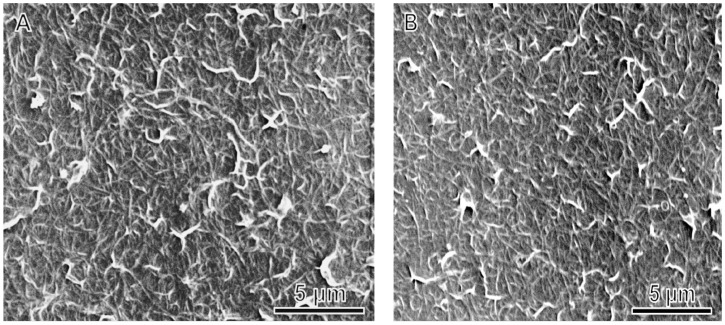

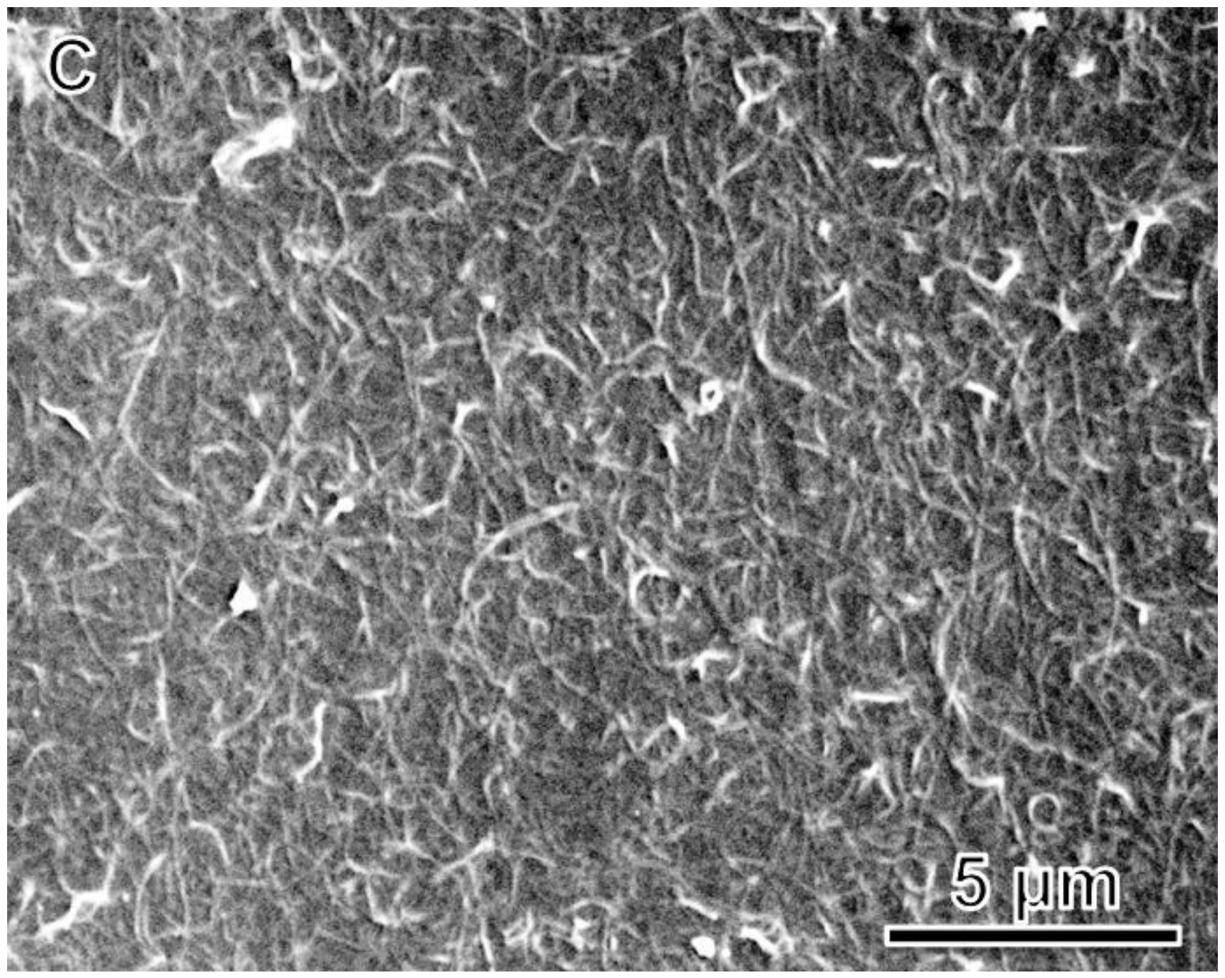
SEM micrographs of rGO-SWNT films prepared from inks with different GO to SWNT mass ratios: (**A**) 5:1, (**B**) 10:1 and (**C**) 20:1.

A large GO to SWNT mass ratio ensures a more efficient disentanglement of SWNT bundles, and the relatively large GO concentration renders an ink viscosity appropriate for coating. Rod-coating GO-SWNT ink thus produces uniform, defect-free thin films. SEM images of these films (Figure 5A–C) show that SWNTs are uniformly dispersed and interconnected with each other over a large area, yielding a percolated 2D conducting network throughout the entire film. The very long length of the SWNTs enable the formation of a conductive network at a very low percolation threshold, thus are expected to give a much improved electrical conductivity. With the increase in the GO portion in the ink, SWNT bundles within the film became thinner. This is in good agreement with the TEM observation of SWNT dispersions as shown in Figure 2, implying that the dispersion state of SWNTs is well maintained during the drying process. Even after microwave heating, the distribution of SWNTs is still rather uniform, without any signs of phase separation.

**Figure 6:**
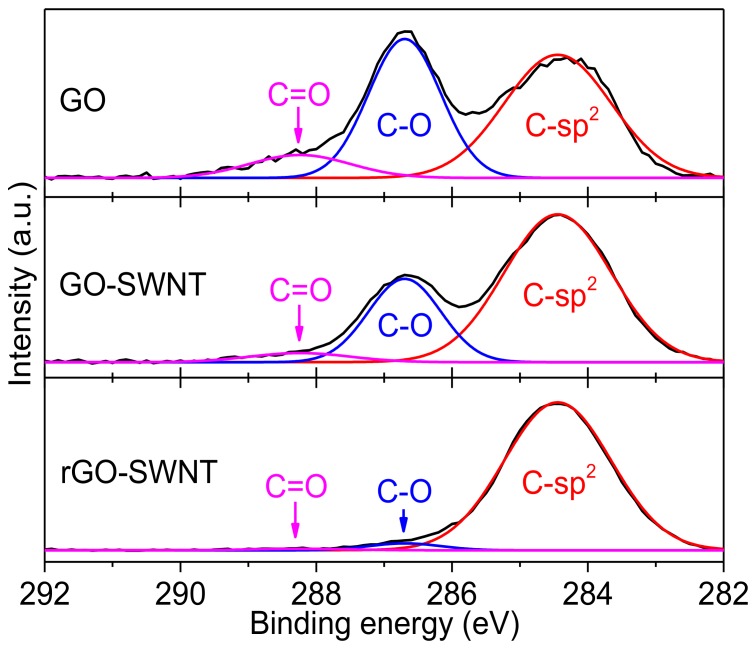
XPS C-1s spectra of GO, GO-SWNT (weight ratio of 10:1) and rGO-SWNT films. Peaks relate to different carbon bonds are indicated.

GO is well known to be nonconductive because of its oxygenated groups. To recover conductivity it must be deoxygenated. Recently, microwave treatment has been shown to be an effective and facile route to remove oxygenated groups from GO and recover its electrical conductivity [32]. Two- to three-second pulses of microwaves can effectively reduce GO into pristine graphene. During the experimental process, we found that microwave treatment of pure GO film (even though it had been treated by hydrazine for ~1 h) did not cause any noticeable change on the film properties, possibly due to the low conductivity of GO, thus leading to poor microwave absorption. In the presence of SWNTs, however, the films heated rapidly to high temperature owing to the strong microwave absorption of SWNTs, which thermally converted GO to rGO. As shown in Figure 6, the resultant rGO-SWNT film contained a negligible amount of oxygen, compared to GO-SWNT film, indicating that the microwave treatment is very efficient in removing the oxygen moieties. The resultant rGO-SWNT films were uniform without disruption or delamination from the substrate.

**Figure 7:**
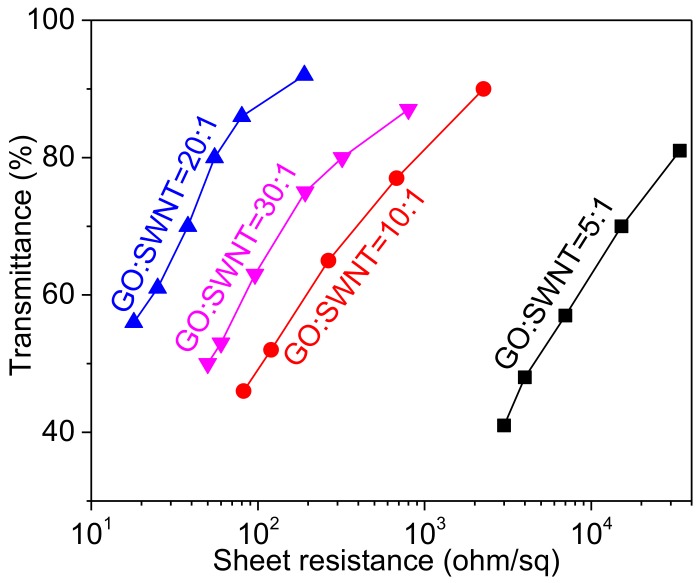
Sheet resistance versus optical transmittance for rGO-SWNT films fabricated from inks with different GO to SWNT mass ratios. The SWNT content in the ink is 0.2 mg/mL.

**Figure 8:**
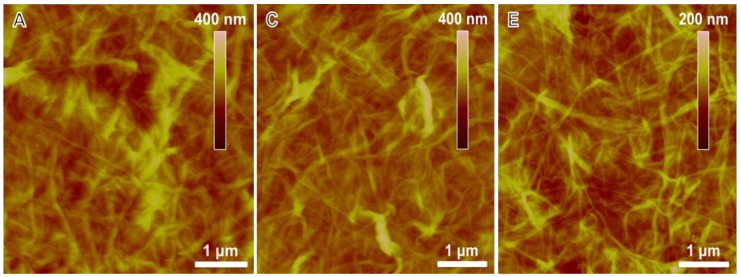

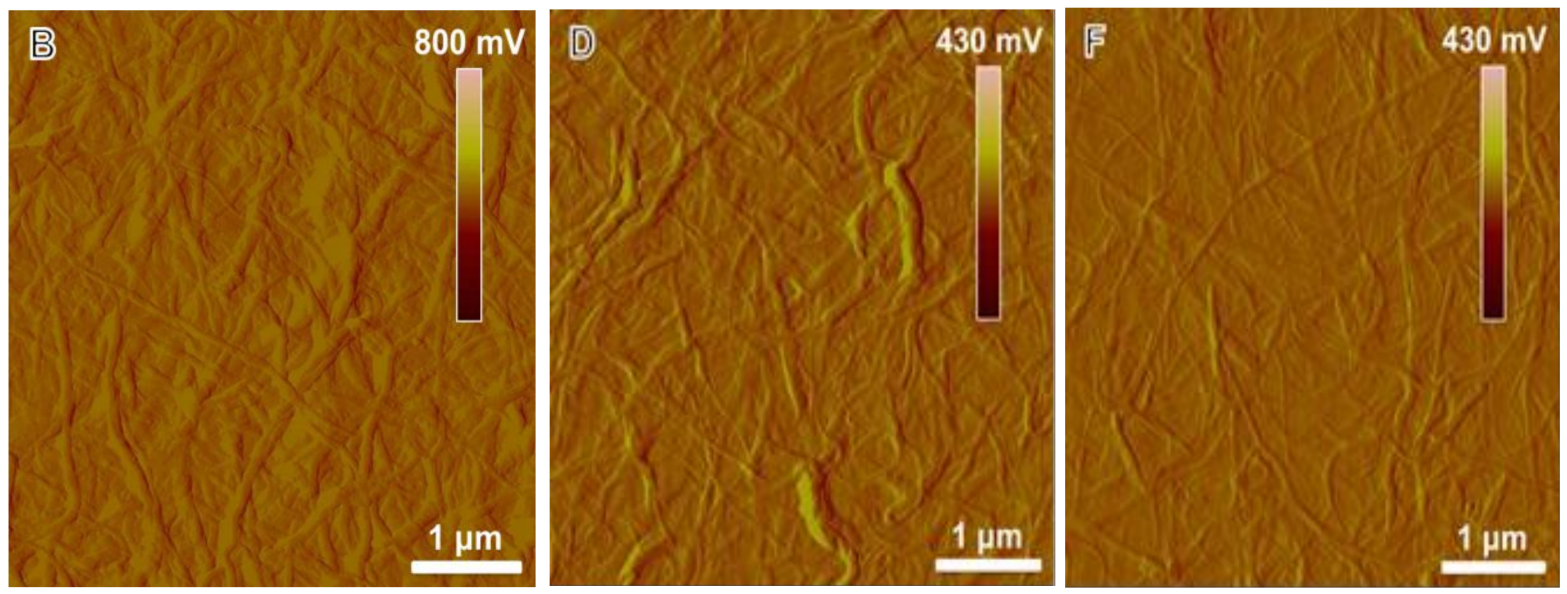
AFM height (**A**, **C** and **E**) and amplitude (**B**, **D** and **F**) microgrqaphs of rGO-SWNT films prepared from inks with different GO to SWNT mass ratios: (**A**) 5:1, (**B**) 10:1 and (**C**) 20:1.

Figure 7 shows that thin films fabricated from various inks exhibit different electro-optical properties. This can be explained by the electrical contacts between nanostructured elements within the films. As shown in Figure 2, inks with lower GO to SWNT mass ratios have thicker SWNT bundles, leading to limited electrical contacts among the SWNT bundles within the rGO-SWNT films. This results in a relatively larger sheet resistance for these films. Incorporation of more GO leads to exfoliation of SWNT bundles, thus increases the number of electrical contacts within the films and reduces the sheet resistance. AFM images in Figure 8 show that an increase in the GO content in the inks leads to thinner SWNT bundles, rendering the film more efficient in transport charge carriers. Besides, these thinner bundles would result in a more smooth film surface (Figure S5), which is preferred for real applications such as organic photovoltaics or OLEDs. At an even larger GO to SWNT mass ratio (~30), the SWNT bundles would not be further exfoliated (Figure S6A,B and Figure 2G). In this case, the electrical contacts between SWNT bundles would be disturbed by rGO sheets, which account for a large portion of the film are less conductive than SWNTs. This results in an increase in the film resistance.

**Figure 9:**
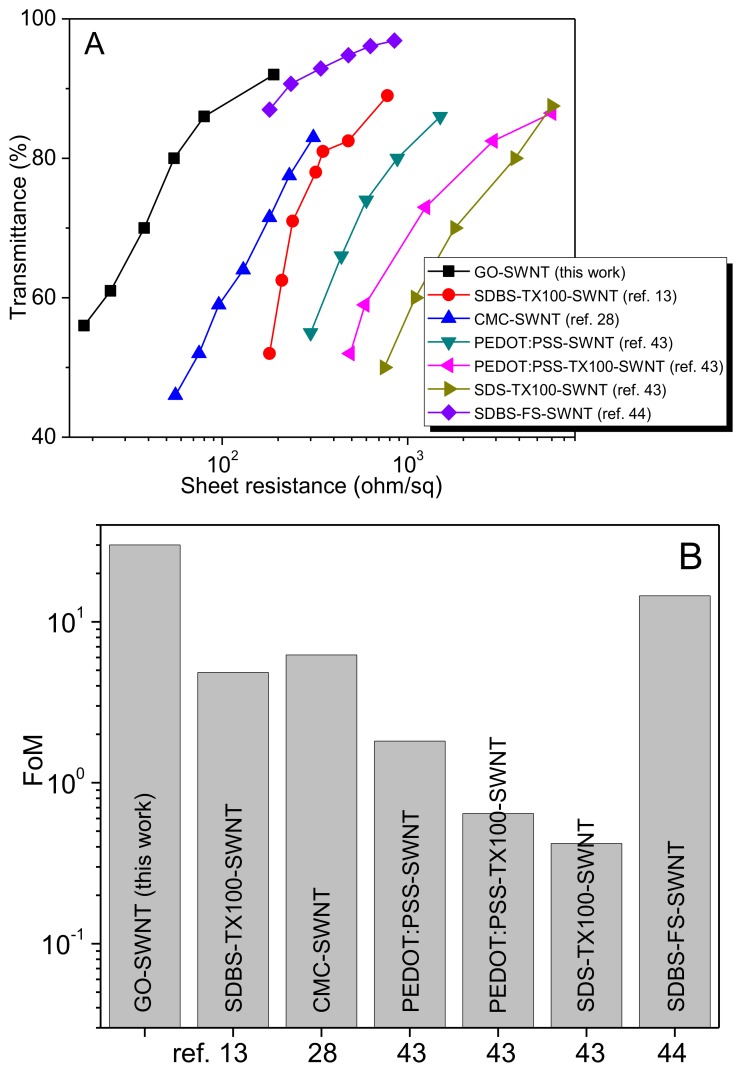
Comparison of electrical properties of rGO-SWNT film with those films contained other dispersing agents. (**A**) Transmittance versus sheet resistance profiles; (**B**) Comparison of figure of merit (FoM) CMC: sodium carboxymethyl cellulose; SDS: sodium dodecyl sulphate; FS: fluorosurfactant (FC-4430).

For TCs, the relationship between transmittance and sheet resistance is generally described by [1]:

(1) $$T=\;(1\;+\;\frac{Z_{0}}{{2R}_{s}}\frac{\sigma_{\mathrm{op}}}{\sigma_{\mathrm{dc}}})$$

where *Z*_0_ is the impedance of free space (377 Ω), *R*_s_ the sheet resistance, σ_op_ the optical conductivity, and σ_dc_ the DC conductivity. The performance of TCs can be evaluated by a parameter, i.e., figure of merit (FoM) given by σ_dc_/σ_op_. FoM offers direct performance comparison between the TCs prepared from various materials or methods over entire range of optical transmittance. Generally, TCs should have low sheet resistance and high optical transmittance, i.e., a large FoM. In Figure 9A we compare the performance of rGO-SWNT film with that of SWNT films prepared using surfactant/polymer as the dispersant. FoMs are determined from the sheet resistance at approximately 75~85% transmittance (Figure 9B). This is because most reported transmittance values of SWNT films fall in this range. It can be seen that the films fabricated from the GO-SWNT system outperform other systems in terms of electro-optical properties. For thin films fabricated from the inks with non-conductive dispersants, the incorporation of dispersants usually results in inferior nanotube-nanotube electrical contacts, giving rise to the resultant film with a large sheet resistance. In contrast, GO sheets are beneficial for the electrical transport of nanotube films. As mentioned before, there exists strong π-π interactions between the GO sheets and SWNTs. After converting to rGO, these conductive sheets serve as nano-“patches” that fixed or repaired SWNT networks by filling their void sites. Additional conducting channels would therefore be created and significantly reduce the film resistance. Another reason for the reduced sheet resistance is mechanical factor in origin. Due to the increased nonpolar nature of rGO compared to GO sheets, the rGO-SWNT interactions should be much stronger than the GO-SWNT interactions. Microwave irradiation of GO-SWNT to yield rGO-SWNT causes film densification. This force of densification is expected to compress SWNT networks, reduce the air gap within the film as well as the nanotube-nanotube junction resistance, and consequently decreases the overall resistance. Since rGO is almost transparent, film transparency of rGO-SWNT remains unchanged as expected. It is noted that the electrical conductivity of rGO-SWNT films can be further enhanced by chemical doping [46]. Simply dipping the films in nitric acid (12M) for 2 min causes a marked decrease in sheet resistance, with a negligible effect on the film transparency (Figure S7A). The average sheet resistance for a ~86% transparent film before doping was ~80 Ω/sq, and decreased to ~25 Ω/sq after doping, demonstrating a three-fold decrease in the equivalent sheet resistance. However, the doping effect does not last very long (Figure S7B), because nitric acid molecules are weakly adsorbed on the nanotube surfaces and can desorb. A poly(3,4-ethylenedioxythiophene):poly-(styrene sulfonate) (PEDOT:PSS) layer has been shown to effectively inhibit the increase of film resistance [47].

As mentioned above, we have used Mayer rod coating to deposit rGO-MWNT films onto glass substrates [14]. The rGO-MWNT film exhibits a sheet resistance of 380 Ω/sq at 85% transmittance. Moreover, the rGO-MWNT film has a FOM value of 6. In contrast, the sheet resistance of rGO-SWNT film in this study is ~80 Ω/sq at ~86% transmittance before doping, and reduces to ~25 Ω/sq after doping. The FOM value of rGO-SWNT film is 30 (Figure 9), being much higher than that of rGO-MWNT sample. Apparently, rGO-MWNT films exhibit much higher sheet resistance and poorer electro-optical performance than rGO-MWNT films in this work. This can be attributed to each graphene layer of MWNTs contributes to the light absorption, resulting in strong light absorption of rGO-MWNT films [30]. In addition, rGO-MWNT films are deposited on rigid glass substrates, so their mechanical flexibility cannot be evaluated through mechanical bending test. On the contrary, rGO-SWNT films are coated on flexible PET plastic substrates, so their electro-optical performance due to mechanical bending can be determined accordingly.

**Figure 10:**
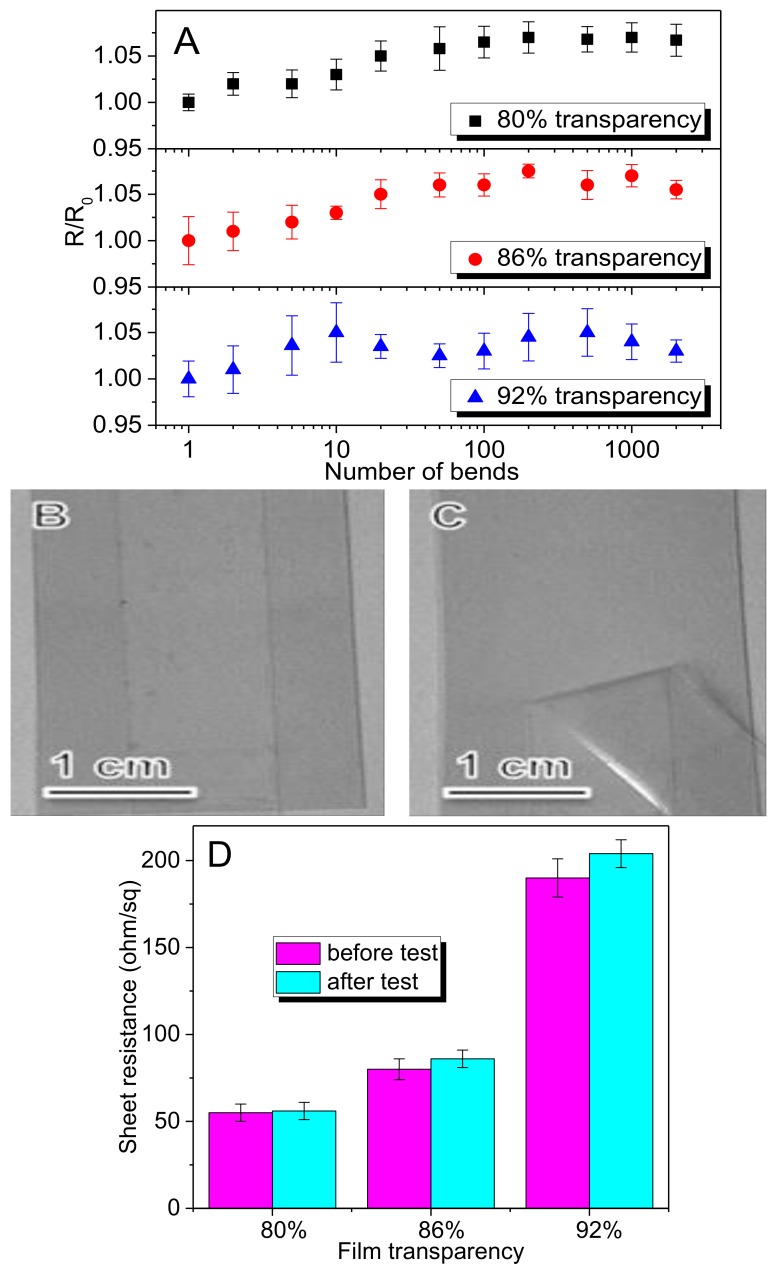
(**A**) Relative sheet resistance vs. benching cycles for rGO-SWNT films. R_0_ is the electrical resistance of samples before bending, and R is the electrical resistance that changes with bending cycles. Error bars reflect standard deviation for five measurements; (**B**,**C**) Mechanical stability of rGO-SWNT/PET film: before and after the Scotch tape test; (**D**) Sheet resistance vs. transparency before and after Scotch tape peeling test.

In addition to excellent electro-optical properties of rGO-SWNT films, TCs require mechanical durability to withstand mechanical bending of flexible, portable consumer electronics. Figure 10A shows that the electrical resistance of the rGO-SWNT films exhibits negligible change over 2000 bending cycles to a radius of 2 mm, with a minor increase in resistance consistent with previous reports, verifying the utility of this material for devices in which a high tolerance to the twist of flexibility is necessary. While the rGO-SWNT exhibits no degradation, PET substrate exhibits plastic deformation under these conditions, which is the main reason contributing to the resistance increase.

While bending stability confirms that the material can deform without failure, it does not necessarily imply strong film adhesion to the substrate. It is known that solution-processed CNT film interacts weakly with the polymer substrate, resulting in poor adhesion of the coating. This can cause significant yield loss during device processing. A scotch tape test was therefore performed to assess the substrate adhesion performance of rGO-SWNT films. A strip of tape is applied to the films and peeled off in a controlled manner. Then the tape and sample are inspected for the film damage. The optical pictures of rGO-SWNT film coated on PET substrate before and after tape testing are shown in Figure 10B,C. No significant film damage is found in the rGO-SWNT film after tape testing. Electrical measurements agree reasonably with this result. Specifically, these films exhibit negligible resistance change after tape adhesion test (Figure 10D). We propose that the microwave treatment plays a critical role in enhancing the film adhesion. As reported before, CNT-polymer welding is an important effect caused by microwave heating [48,49,50,51,52]. It usually leads to strong adhesion between the nanotubes and the plastic. Given microwave heating is highly selective, only nanotubes absorb microwave energy and heated accordingly, so it can be carried out without causing significant distortion of underlying PET substrate [49].

It can be concluded that the rGO-SWNT samples exhibit low sheet resistance of ~80 Ω/sq and ~25 Ω/sq respectively at ~86% transmittance before and after nitric acid doping. The rGO-SWNT film also exhibits a high FOM value of 30. The excellent electro-optical properties, coupled with the intrinsic flexibility and good film adhesion property make the rGO-SWNT conductor an excellent or ideal replacement for ITO. In terms of scalable film production and high quality of the products, the method introduced in this study is much superior or better than those films reported in the literature. In Figure S8A we show a film of ~4 × 5 inch^2^ prepared by this method. The film appears to be very uniform without any nanotube aggregates. Moreover, microscopic examinations of different locations of the film also reveal a similar morphology (Figure S8B,C). Therefore, it is expected that the GO-SWNT ink can be well adapted to the coating processes in industries including slot, slide, and roll-to-roll, facilitating a fast and mass production of continuous film with high quality. In addition, the film properties can be fine-tuned by monitoring the compositions and/or the thicknesses of the films. We anticipate that this work can trigger further study of novel dispersants for SWNTs and the better use of resulting SWNT inks with functional properties.

## 4. Conclusions

We have presented a promising graphene oxide-based approach for preparing carbon nanotube inks for scalable production of carbon-based TCs. This strategy combines scalable, low-cost production of graphene oxide with tunable nanotube ink formulation for a technique compatible with large-scale production, thus providing new opportunities for broad integration of the films in industrial applications. The resulting films exhibit many of the desirable properties such as good film adhesion, sheet resistance of ~80 Ω/sq at 86% transparency, being the best amongst solution-processed carbonaceous films. Moreover, nitric acid doping can further decrease the sheet resistance to ~25 Ω/sq. Finally, microwave treatment induces rapid welding between the film and the substrate, yielding the films resilient to strong mechanical stresses including cyclic bending and adhesion testing. The concurrent realization of these properties in a scalable and adaptable process represents a significant advance for using nanotube inks in manufacturing transparent conductors.

## Figures and Tables

**Table 1:** Time parameters for fabricating TCs using GO-SWNT inks.

Ink Composition	*t*_level_ (s)	*t*_dry_ (s)	*t*_dewet_ (s)
0.2 mg/mL SWNT + 1 mg/mL GO	8.33 × 10^−4^	600	3.32 × 10^3^
0.2 mg/mL SWNT + 2 mg/mL GO	1.10 × 10^−3^	500	6.16 × 10^3^
0.2 mg/mL SWNT + 4 mg/mL GO	1.41 × 10^−3^	400	1.12 × 10^4^
0.2 mg/mL SWNT + 6 mg/mL GO	1.83 × 10^−3^	300	2.05 × 10^4^

where *t*_level_ was calculated using high-shear (~50 s^−1^) viscosity; *t*_dewet_ was determined with low-shear (~0.1 s^−1^) viscosity; and *t*_dry_ was estimated by recording the time taken to cause gelation in the coated films. The diameter of wire coiled on the rod was ~0.6 mm. A length scale (*l*) of 2 mm (~10% of width of the coated film) was used for calculating *t*_dewet_. The wet coating thickness and wire diameter are assumed to be 1:10 [46].

## Supplementary Materials

The following are available online at http://www.mdpi.com/2079-4991/8/4/224/s1. Figure S1: TEM micrograph of as-received SWNTs. Figure S2: AFM image of as-synthesized GOs (on freshly cleaved mica). Figure S3: Contact angle results for GO-SWNT inks with different compositions on PET substrates. (A) 1 mg/mL, (B) 2 mg/mL, (C) 3 mg/mL, (D) 4 mg/mL and (E) 6 mg/mL. The SWNT content is 0.2 mg/mL. Contact angles (from left to right) are 59.7°, 54.4°, 59.9°, 56.4° and 55.6°, respectively. Figure S4: Contact angle results for GO-SWNT inks with different compositions on PET substrates after oxygen plasma treatment. (A) 1 mg/mL, (B) 2 mg/mL, (C) 3 mg/mL, (D) 4 mg/mL and (E) 6 mg/mL. The SWNT content is 0.2 mg/mL. Contact angles (from left to right) are 10.2°, 10.5°, 10.7°, 10.7° and 10.6°, respectively. Figure S5: 3D topography of rGO-SWNT films prepared from different GO-SWNT dispersions: (A) 5:1, (B) 10:1 and (C) 20:1. Figure S6: (A) AFM height and (b) amplitude micrographs of rGO-SWNT film prepared from the ink having GO to SWNT mass ratio of 30:1. Figure S7: (A) Effect of nitric acid doping on electro-optical performance of 86% transparent rGO-SWNT films. (B) Stability of nitric acid-doped 86% transparent rGO-SWNT film. Figure S8: (A) Photograph of a GO-SWNT film of ~4 × 5 inch^2^ prepared from GO-SWNT ink by rod coating. (B) and (C) Low magnification SEM micrographs of location I and II showing morphological uniformity of the film. The white dots are catalyst impurities.
