# Supplementary material for: Scalable Fabrication of High-Performance Transparent Conductors Using Graphene Oxide-Stabilized Single-Walled Carbon Nanotube Inks
Source: Nanomaterials (Basel). 2018 Apr 7;8(4):224. doi: 10.3390/nano8040224 (PMC5923554; doi:10.3390/nano8040224)
Supplement: Supplementary file 1 [file nanomaterials-08-00224-s001.doc]

Supplementary Information

Scalable fabrication of high-performance transparent conductors using graphene oxide-stabilized single-walled carbon nanotube inks

Linxiang He^1^, Chengzhu Liao^2^,* and Sie Chin Tjong^1^,*


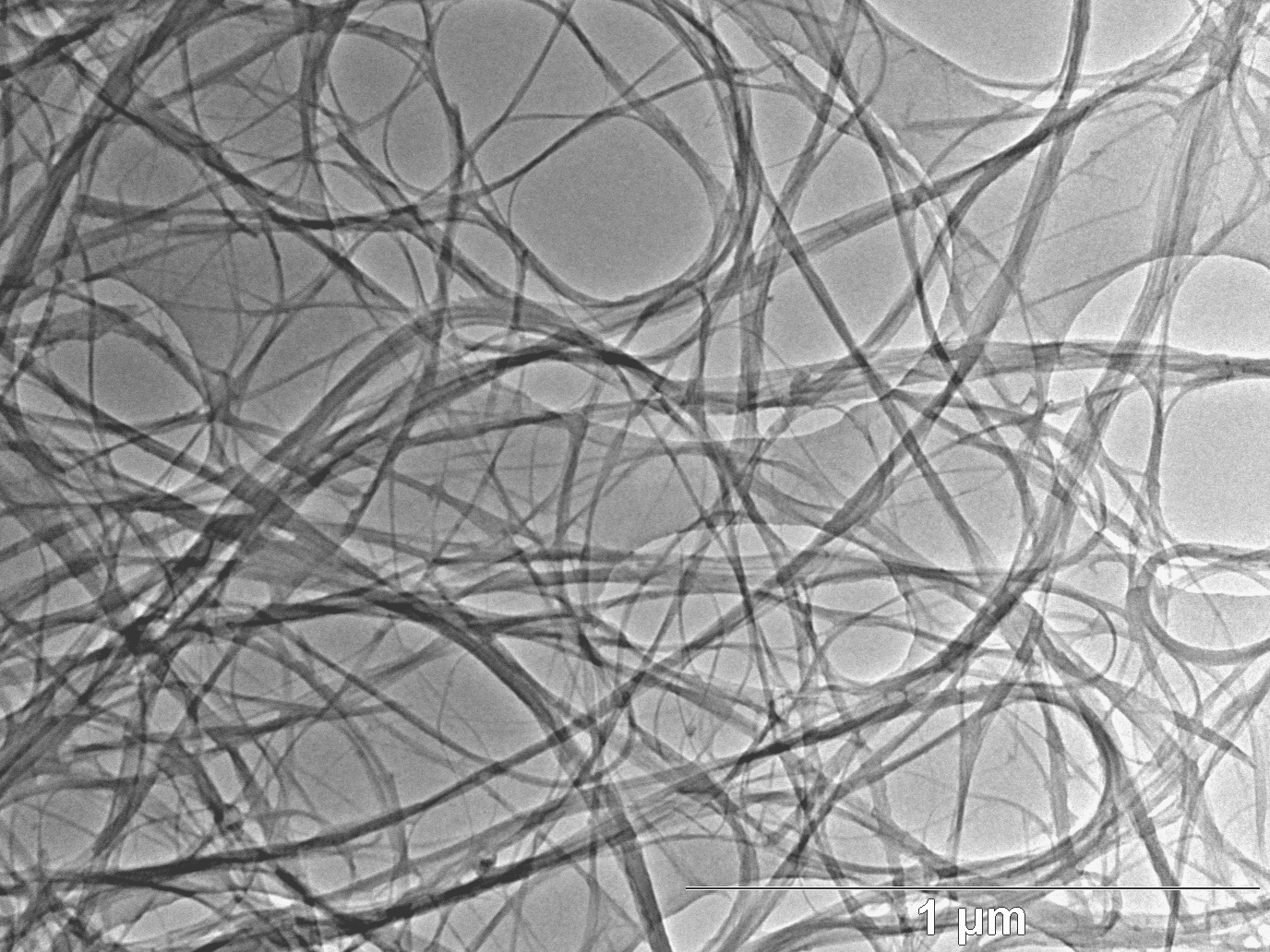


**Figure S1**. TEM micrograph of as-received SWNTs.


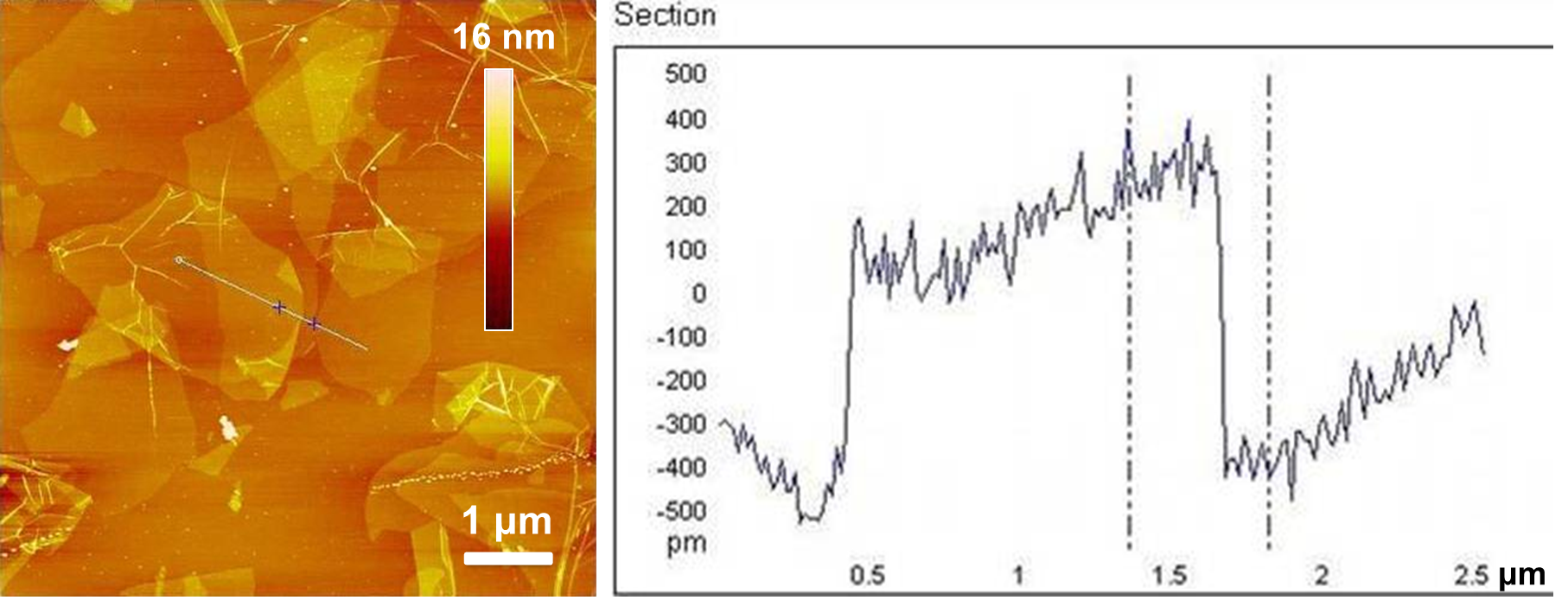


**Figure S2**. AFM image of as-synthesized GOs (on freshly cleaved mica).


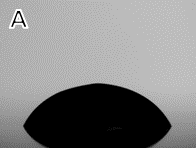

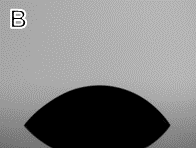

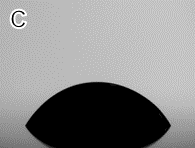

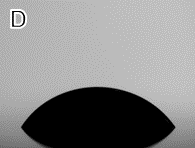

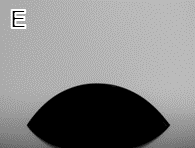


**Figure S3**. Contact angle results for GO-SWNT inks with different compositions on PET substrates. (A) 1 mg/mL, (B) 2 mg/mL, (C) 3 mg/mL, (D) 4 mg/mL and (E) 6 mg/mL. The SWNT content is 0.2 mg/mL. Contact angles (from left to right) are 59.7°, 54.4°, 59.9°, 56.4° and 55.6°, respectively.


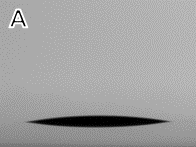

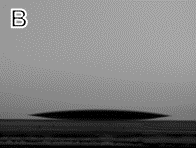

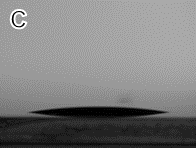

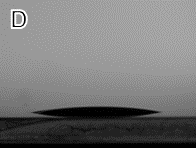

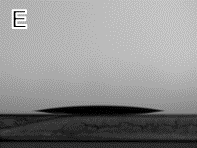


**Figure S4**. Contact angle results for GO-SWNT inks with different compositions on PET substrates after oxygen plasma treatment. (A) 1 mg/mL, (B) 2 mg/mL, (C) 3 mg/mL, (D) 4 mg/mL and (E) 6 mg/mL. The SWNT content is 0.2 mg/mL. Contact angles (from left to right) are 10.2°, 10.5°, 10.7°, 10.7° and 10.6°, respectively.


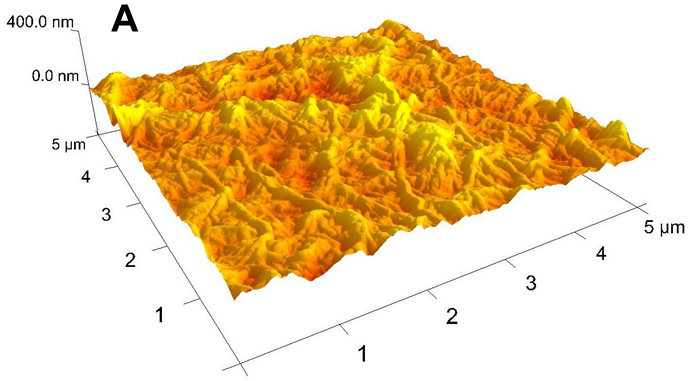


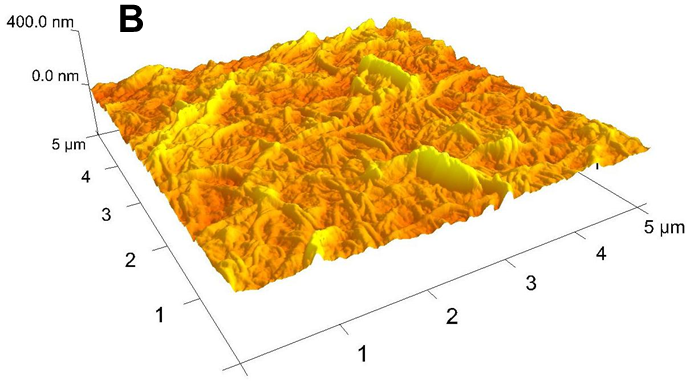


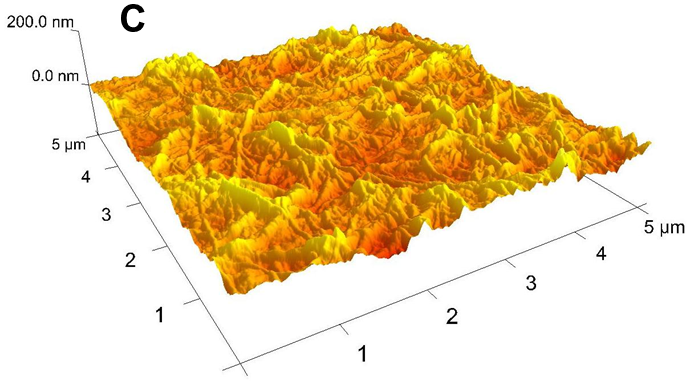


**Figure S5**. 3D topography of rGO-SWNT films prepared from different GO-SWNT dispersions: (A) 5:1, (B) 10:1 and (C) 20:1.


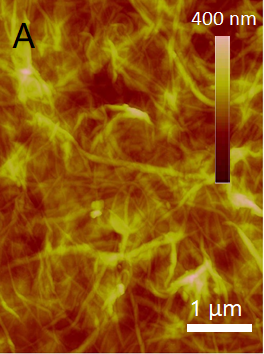

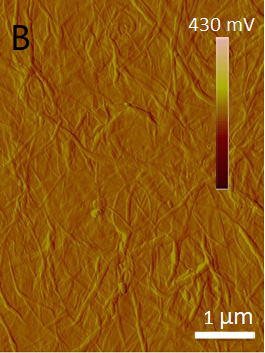


**Figure S6**. (A) AFM height and (b) amplitude micrographs of rGO-SWNT film prepared from the ink having GO to SWNT mass ratio of 30:1.

**Figure S7**. (A) Effect of nitric acid doping on electro-optical performance of 86% transparent rGO-SWNT films. (B) Stability of nitric acid-doped 86% transparent rGO-SWNT film.


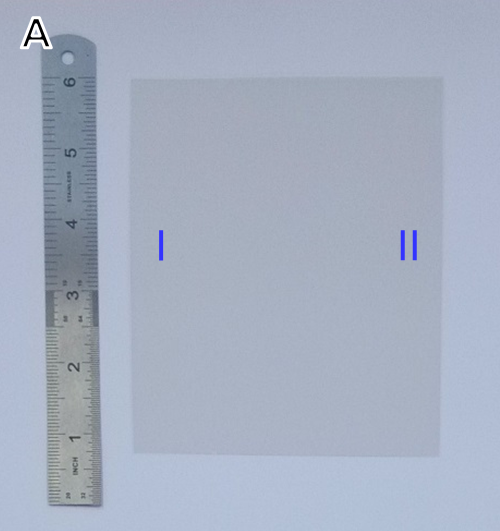


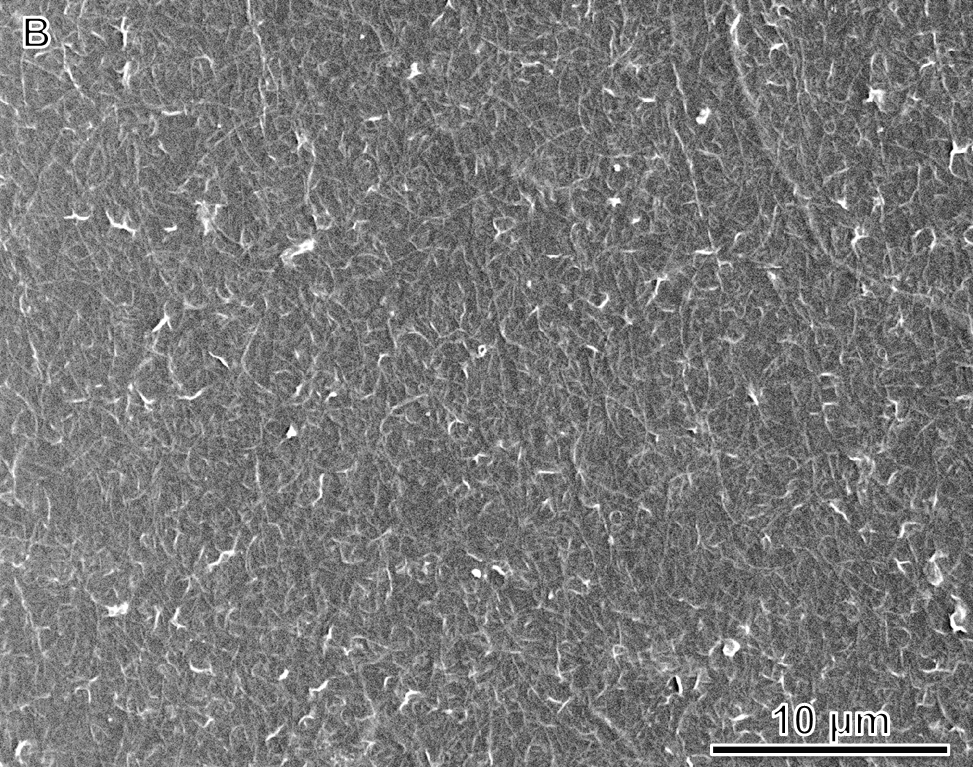


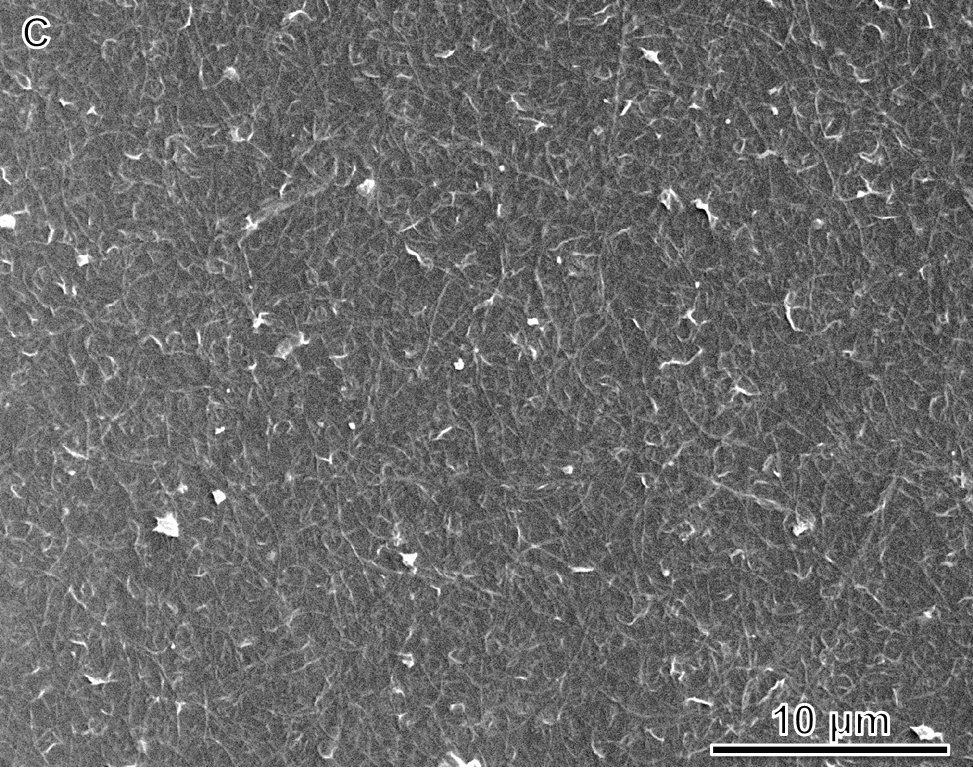


**Figure S8**. (A) Photograph of a GO-SWNT film of ~ 4 × 5 inch^2^ prepared from GO-SWNT ink by rod coating. (B) and (C) Low magnification SEM micrographs of location I and II showing morphological uniformity of the film. The white dots are catalyst impurities.
